# Hippocampal atrophy and verbal episodic memory performance in amnestic mild cognitive impairment and mild Alzheimer’s disease: A preliminary study

**DOI:** 10.1590/S1980-57642009DN20100008

**Published:** 2008

**Authors:** Nathalia Carollina Peruzza Marchiani, Marcio Luiz Figueredo Balthazar, Fernando Cendes, Benito Pereira Damasceno

**Affiliations:** 1 Graduate student. Department of Neurology; Medical School, State University of Campinas (UNICAMP).; 2 MD, PhD student. Department of Neurology; Medical School, State University of Campinas (UNICAMP).; 3 MD, PhD, Associate Professor. Department of Neurology; Medical School, State University of Campinas (UNICAMP).; 4 MD, PhD, Professor. Department of Neurology; Medical School, State University of Campinas (UNICAMP).

**Keywords:** hippocampal atrophy, MRI, memory, Alzheimer’s disease, mild cognitive impairment, atrofia hipocampal, MRI, memória, doença de Alzheimer, transtorno cognitivo leve

## Abstract

To evaluate hippocampal volume in patients with AD and aMCI, and correlate its atrophy with verbal episodic memory performance.

**Methods:**

We studied 42 individuals older than 50 years, including 14 with amnestic mild cognitive impairment (aMCI), 14 with mild Alzheimer’s disease (AD) and 14 normal controls. All individuals were submitted to the Rey auditory verbal learning test (RAVLT) to evaluate episodic memory. They were also submitted to the forward (FDS) and backward digit span (BDS) subtest of WAIS-R to evaluate working memory and attention, and to the Mini Mental State Examination (MMSE). Hippocampal volumetric measurements were performed according to anatomic guidelines from a standard protocol using high-resolution T1-inversion recovery 3-mm coronal MRI slices. Hippocampal volumes (HV) were corrected for the variation in total intracranial volume. There was no significant difference between the three groups concerning age and education.

**Results:**

On RAVLT, there was a continuum between the three groups, with AD recalling less words, controls more, and aMCI subjects showing an intermediate performance on all sub-items. We found an asymmetry between HVs, with smaller mean left HV for all groups. ANOVA and post hoc Tukey’s test for comparisons of HV showed a significant difference among groups, with difference between controls and both AD and aMCI, although there was no significant difference between AD and aMCI groups.

**Conclusions:**

There was a significant correlation between hippocampal volumes and scores on RAVLT, confirming that medial temporal structures are closely associated with memory performance in normal ageing as well as in aMCI and AD.

## Abstract (pt)

Avaliar os volumes hipocampais (VH) em pacientes com doença de Alzheimer (DA) leve e comprometimento cognitivo leve amnéstico (aCCL).

**Métodos:**

Estudamos 42 sujeitos maiores de 50 anos, incluindo 14 com DA leve, 14 com aCCL e 14 controles. Todos foram submetidos ao teste de aprendizado auditivo verbal de Rey (TAAVR) para avaliação de memória episódica, ao teste de extensão de dígitos direto e indireto do WAIS-R, para avaliação de memória operacional e atenção e ao Mini Exame do Estado Mental (MEEM). As medidas de volumetria hipocampal foram obtidas de acordo com as diretrizes anatômicas de um protocolo padrão usando imagens coronais ponderadas T1 inversion-recovery de 3 mm. Os VH foram corrigidos para a variação do volume intracraniano total. Não houve diferença significativa entre os 3 grupos quanto a idade e escolaridade.

**Resultados:**

No TAAVR houve um *continuum* no desempenho dos 3 grupos, com os pacientes com DA leve evocando menos palavras, os controles mais e os aCCL mostrando um desempenho intermediário em todos os subitens. Encontramos também uma assimetria entre os VH, com o lado esquerdo menor que o direito nos 3 grupos. O teste de análise de variância (ANOVA) seguido do teste post hoc de Tukey para comparações entre os VH dos grupos mostrou diferença significativa entre controles e DA e aCCL, mas não entre DA e aCCL. Encontramos também correlação estatística significativa entre os VH e o desempenho no TAAVR.

**Conclusões:**

As estruturas do lobo temporal medial estão intimamente ligadas ao desempenho de memória episódica tanto no envelhecimento normal quanto no aCCL e DA.

Memory is a complex psychological function that is closely associated with medial temporal lobe structures. Since the H.M. case described in the early 1950s, it has been known that circumscribed brain lesions within the limbic system may deteriorate the ability to form new memories.^1^

Patients with Alzheimer’s disease (AD) and amnestic mild cognitive impairment (aMCI) show a markedly reduced ability to retain new information: they often have difficulty in recalling appointments, shopping list items, names of people, and perform poorly on verbal episodic memory tests. This memory impairment is the earliest clinical symptom and a prominent feature throughout the course of AD.^2,3^

The hippocampus is a central component of the medial temporal lobe memory system, and its structural integrity is necessary for declarative memory.^1,2^ There are several neuroimaging evidences for loss of hippocampal tissue in human diseases associated with memory impairments, and findings of magnetic resonance imaging (MRI) studies have established that volumetry of the hippocampus is useful in assisting the clinical diagnosis of AD.^4-6^ In patients with aMCI, a condition that is often transitional to AD, hippocampal cortex pathology lies between the values measured in controls and mild AD.^7^

In the present study, our aim was to evaluate hippocampal volume in patients with AD and aMCI, and correlate its atrophy with verbal episodic memory performance.

## Patients and methods

We studied 42 individuals older than 50 years, comprising 14 with aMCI, 14 with mild AD attended at the Unit for Neuropsychology and Neurolinguistics (UNICAMP Clinic Hospital), and 14 normal controls. Routine laboratory examinations for dementia assessment (including B12 and folate dosage, serology for syphilis, thyroid hormones) and brain computed tomography were carried out in all patients. The local ethics committee approved this research.

aMCI in our clinic is a diagnosis carried out by trained neurologists using a standardized mental state battery. The diagnostic process consisted of a detailed interview with the patient and informant. All patients were submitted to the Cambridge Mental Disorders of the Elderly Examination (CAMDEX) which comprises structured interviews with the patient and, separately, with an informant, along with evaluation of patient’s current medical and psychiatric status and family history. Participants were also submitted to the CAMDEX cognitive test battery (CAMCOG), which includes eight subscales: memory, orientation, language, attention, abstract thinking or similarities, calculation and perception.^8^

MCI diagnosis followed the criteria of the International Working Group on Mild Cognitive Impairment,^9^ and was classified as follows:

(i) the person is neither normal or demented;
(ii) there is evidence of cognitive deterioration shown by either objectively measured decline over time and/or subjective self-report of decline and/or by informant in conjunction with objective cognitive deficits; and
(iii) activities of daily living are preserved and complex instrumental functions are either intact or minimally impaired.

We considered a diagnosis of aMCI if the clinical history and cognitive performance pointed to an exclusive memory deficit and Clinical Dementia Rating^10^ score of 0.5, with obligatory memory score of 0.5. This classification was achieved using a semi-structured interview.

For probable AD diagnosis, we used the criteria of the National Institute of Neurological and Communicative Disorders and Stroke (NINCDS) and Alzheimer’s Disease and Related Disorders Association (ADRDA)^11^ including only patients classified as CDR 1. Exclusion criteria were history of other neurological or psychiatric diseases, head injury with loss of consciousness, use of sedative drugs in the last 24 hours before the neuropsychological assessment, drug or alcohol addiction and prior chronic exposure to neurotoxic substances. The control group consisted of subjects with CDR 0 without previous history of neurological or psychiatric disease, or memory complaints.

All individuals were submitted to the Rey auditory verbal learning test (RAVLT)^12^ to evaluate episodic memory, which consists of fifteen words read aloud for five consecutive trials (List A), followed by a free-recall test. We considered immediate memory the mean of these five trials. After the fifth trial, a new interference list of fifteen words is presented (List B) followed by a free-recall test of that list. Soon afterwards, a free-recall of the first list is tested without representation. After a twenty-minute delay period, subjects are again required to recall words from List A (delayed recall). Finally, the patient must identify List A words from a list of fifty words which includes Lists A and B and twenty other words phonemically or semantically related to lists A and B (recognition). They were also submitted to the forward (FDS) and backward digit span (BDS) subtest of WAIS-R^13^ to evaluate working memory and attention, as well as to the Mini Mental State Examination (MMSE).^14^

### MRI volumetry

MRI acquisition was performed on a 2-T scanner (Elscint Prestige^®^, Haifa, Israel), in three orthogonal planes, and a volumetric saggital T1 acquisition for multiplanar reconstruction. Hippocampal volumetric measurements were performed according to anatomic guidelines from a standard protocol^15^ in T1-IR 3-mm coronal slices (flip angle=200º; TR=2800, TE=14, inversion time (TI)=840, matrix 130×256, FOV=16 cm×18 cm). We performed manual delineation of the entire extension of hippocampal formation using the NIH-Image program^®^ (developed at the United States National Institutes of Health and available on the Internet at http://www.rsb.info.nih.gov/nih-image/).

Hippocampal volumes (HV) were corrected for the variation in total intracranial volume, and asymmetry indexes were determined for each subject as the ratio of the smaller to the larger hippocampus. Volumes were transformed into Z scores: number of standard deviations from the mean of control group. Z scores below –2.0 were indicative of atrophy. The investigators who interpreted MRIs and performed MRI volumetric measurements were blinded to patients’ clinical and neuropsychological information.

Data analysis by means of Systat software used ANOVA and a post-hoc Tukey test for group comparisons of demographic, cognitive and volumetric scores. Multiple linear regressions were used to compare RAVLT scores with other relevant variables. Statistical significance considered was p<0.05.

## Results

As shown in Table 1, there was no significant difference between the three groups concerning age [F (3,39)=3.105, p=0.056] and education [F (3,39)=0.196, p=0.822]. On RAVLT, there was a continuum between the three groups, with AD recalling less words, controls more, and aMCI subjects showing an intermediate performance in all subitems (immediate memory, delayed recall and recognition).

**Table 1:** Demographic and neuropsychological data.

	AD (mean±SD)	aMCI (mean±SD)	Controls (mean±SD)
Age	75.07±6.90	68.14±9.75	69.00±7.09
Education	6.14±5.71	6.43±4.54	7.21±3.56
MMSE	22.86±2.74^a^***, ^b^***	26.93±2.59^b^*	29.07±0.73
Delayed recall RAVLT	1.36±1.28^a^***, ^b^***	4.14±2.60^b^**	9.57±3.25
Recognition RAVLT (correct response - false positive)	-1.07±6.33^a^***, ^b^***	4.36±4.55^b^**	11.86±1.88
Immediate memory	5.00±1.12^a^***, ^b^***	7.01±1.41^b^**	9.86±1.33
FDS	4.50±1.09	4.50±0.76	4.93±0.73
BDS	3.21±0.80^b^*	3.14±1.03^b^*	4.14±1.10

a Significantly different to aMCIs;

b Significantly different to controls;

*** p<0.0001;

** p<0.001;

* p<0.05

We found an asymmetry between HVs, with smaller mean left HV for all groups (Table 2). ANOVA and *post hoc* pairwise comparisons of hippocampal volumes using Tukey’s test, showed a significant difference among groups, with difference between controls, AD and aMCI (ANOVA; p<0.00001), although there was no difference between AD and aMCI groups (Table 2).

**Table 2:** Hippocampal volume (mm3).

	AD (mean±SD)	aMCI (mean±SD)	Controls (mean±SD)
	2545.18±433.49^a^***	2720.05±291.94^a^***	3245.14±266.31
Left hippocampus	2406.07±410.89^a^***	2550.41±294.87^a^***	3058.03±217.93

a Significantly different to controls;

*** p<0.0001

Multiple regression analysis including hippocampal volumes from all subjects (AD, aMCI and controls) as independent variables and RAVLT, FDS, BDS and MMSE as dependent variables, showed a significant relationship between volumes and scores on RAVLT subitems and MMSE (p<0.00001). Pearson’s correlation coefficients for left and right hippocampal volumes and each test are shown in Table 3.

**Table 3:** Pearson's correlation coefficients (r) for left and right hippocampal volumes and each test.

Test	Right HV	Left HV
MMSE	0.62	0.62
FDS	0.22	0.16
BDS	0.35	0.27
Delayed recall RAVLT	0.66	0.65
Recognition RAVLT	0.51	0.51

## Discussion

Our results tended to confirm previous studies in which AD patients had a smaller HV compared to normal controls, while aMCI patients had intermediate atrophy (though not statistically significant in our sample). This finding is in accordance with neuropathological studies in which aMCI subjects showed an intermediate pattern of neurofibrillary changes of aging and pathologic features of very early AD, since they showed neurofibrillary tangles in the entorhinal cortex and hippocampal formation.^7,16^ One possible reason for the fact that we did not find significant hippocampal volume differences between mild AD and aMCI HV is the clinical proximity between these two clinical entities and their close pathological relationship. Petersen et al showed that neuropathologists often characterized MCI cases as having prodromal or incipient AD, meaning that they did not fulfill the criteria for AD but were suggestive of being in transition (diffuse amyloid in the neocortex and frequent neurofibrillary tangles in medial temporal lobe structures).^7^ In all groups, there was an asymmetry among left (more atrophic) and right hippocampus, a fact that is in disagreement with other studies, where a right-greater-than-left asymmetry is seen in normal controls, but is in accordance with other papers where MCI cases may present a reversal of this normal hippocampal asymmetry.^4,17,18^

We found a correlation between episodic memory and right and left HVs, confirming that quantitative assessment of medial temporal structures may serve as a surrogate marker of memory performance in normal ageing as well as in AD.^6,19,20^ Measurement of other medial temporal structures such as amigdala, parahippocampal formation, entorhinal and perirhinal cortices, as well as regional hippocampal shape differences (head versus body, for example) may help further in differentiating mild cognitive impairment from initial stages of AD.^1,18,19,21^ Some authors have shown that hippocampal and entorhinal cortex volumes can contribute to the prediction of MCI conversion to AD, although cognitive tests provide better accuracy.^22,23^ Attention may have influenced delayed recall performance in the AD group, since there was significant correlation with the BDS.

In conclusion, our preliminary findings show that there is a significant HV difference between AD, aMCI and controls, but not between AD and MCI; the 3 groups showed more left than right hippocampal atrophy; and episodic memory correlated with left and right HV. Our study had some limitations including the small sample size and the fact that AD patients were older than MCI patients and controls where this approached statistical significance (p= 0.056). Further studies employing larger sample of patients and controls as well as measures of other medial temporal structures are needed to reach definitive conclusions.

## Figures and Tables

**Figure 1:**
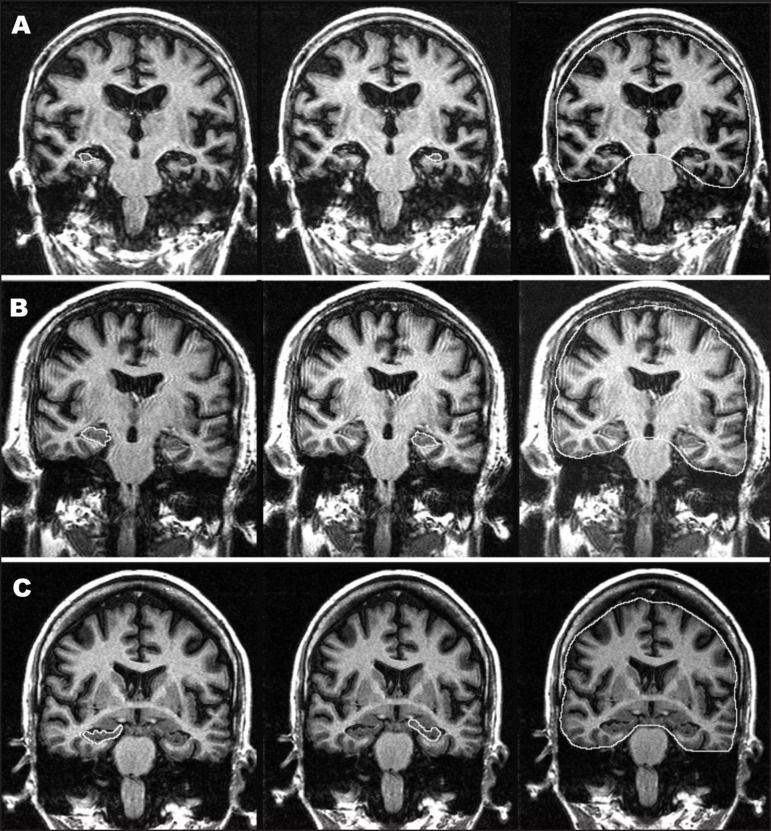
Illustrative pictures of T1-IR coronal slice delineation of the entire extension of right and left hippocampal formation and intracranial volume. (A) Mild AD; (B) aMCI; (C) Normal controls.
